# Dentist-reported differences in services provided to patients with public vs. private dental insurance

**DOI:** 10.1186/s12903-023-03134-4

**Published:** 2023-06-29

**Authors:** Julie Reynolds, Aparna Ingleshwar, Pamela Nwachukwu, Susan McKernan, Peter Damiano

**Affiliations:** 1 University of Iowa College of Dentistry, 221 South Quad, 310 S. Grand Ave., Iowa City, IA 52242 USA; 2 University of Minnesota School of Dentistry, Minneapolis, USA

**Keywords:** Medicaid, Dentistry, Access to care, Private insurance, Procedure type

## Abstract

**Introduction:**

Variation in dentists’ provision of types of dental services based on patients’ insurance may impact population access to comprehensive care. The aim of this study was to describe differences in the types of services provided to adult patients with Medicaid versus private insurance among private practice general dentists.

**Methods:**

The data source was a 2019 survey of private practice dentists in Iowa, and the study sample included general dentists with current or recent participation in Iowa’s Medicaid program for adults (n = 264). Bivariate analyses were used to compare differences in the types of services provided to privately insured and publicly insured patients.

**Results:**

Dentists reported the greatest differences in services provided to patients with public versus private insurance for prosthodontic procedures, including complete dentures, removable partial dentures, and crown and bridge services. Endodontic services were the least frequently provided category of services provided by dentists for both patient groups. Patterns were generally similar among both urban and rural providers.

**Conclusion:**

Access to dental care for Medicaid members should be evaluated not only on the proportion of dentists who see new Medicaid patients but also on the types of services dentists provide to this population.

## Introduction

Within the practice of general dentistry, there is significant variation in the types of procedures that dentists provide themselves and which procedures they refer to another provider. Decisions about procedure provision are usually made at the individual provider level, and are based on provider training, practice infrastructure and staffing, as well as their personal comfort and/or interest in doing certain procedures. However, these individual-level decisions may impact population access to comprehensive dental care if a large segment of general dentists do not perform certain procedures that would be considered part of comprehensive care, and access to specialists are not readily available. For example, one study found that 38% of general dentists do not perform endodontic therapy on molar teeth [1].

In addition to provider comfort and interest influencing procedure provision, patients’ type of dental insurance (e.g., public or private) may also have an impact, given varying levels of coverage, reimbursement, and cost sharing. To the authors’ knowledge, only one study has examined dentist-reported differences in dental services offered to patients based on insurance type. The study examined how often dentists completed certain procedures using questionnaire data from the National Practice-Based Research Network of general dentists, and found that dentists who had more publicly insured patients in their practice completed fewer esthetic procedures and implants than dentists who had fewer publicly insured patients [1].

Several studies have examined differences in population-based procedure mix by payer type using claims data or patient-reported national survey data, and found a comparable service mix among publicly and privately insured children, but a lower share of preventive services among publicly insured adults compared to privately insured [2, 3]. These studies measure the types of services *received*, and it is not known to what degree these differences are driven by disparities in disease burden and treatment need as opposed to differences in the types of dental services *offered* to patients with public versus private insurance.

Medicaid reimbursement may be a key driver of potential differences in the types of services offered to publicly versus privately insured patients. On average, states that provide dental benefits for Medicaid-enrolled adults reimburse approximately half of what private dental insurance pays; among the 31 states with available data that provided extensive or limited dental benefits to Medicaid-enrolled adults as of 2020, reimbursement rates as a percentage of private dental insurance reimbursement ranged from 31 to 87% [4]. For some types of services, such as costly prosthodontic services that usually incur a lab fee, Medicaid reimbursement may not cover the actual or marginal cost of providing care. In this scenario, dentists who participate in Medicaid may elect to limit services in order to minimize financial loss related to cost relative to reimbursement. Dentists have been observed to limit their Medicaid participation in other ways, such as only accepting a set number of patients per month or only their own patients who transition to Medicaid [5]. It is not known to what degree dentists limit the types of services provided to their Medicaid patients.

Given the paucity of literature on the types of services provided by dentists, and variation by insurance type, the aim of this study was to describe differences in the types of services provided to patients with Medicaid versus private insurance by private practice general dentists in the state of Iowa. At the time of the survey, the state provided comprehensive dental benefits to Medicaid-enrolled adults and Medicaid reimbursement was approximately 48% relative to private insurance reimbursement [4].

## Methods

The data source was a 35-item survey administered in April 2019 by mail to all dentists in private practice in the state of Iowa (n = 1,287). Survey topics included provider participation in the state Medicaid program for adults, called the Dental Wellness Plan (DWP); DWP-related attitudes and experiences; procedures provided to patients by insurance type; and practice-related information. Mailing address and dentist demographic information were accessed from the Iowa Dentist Tracking System, which tracks state dentist workforce information and is part of the University of Iowa’s Office of Statewide Clinical Education Programs [6]. A reminder postcard was sent 1 week after mailing, and a second survey was sent to nonrespondents 2 weeks after the postcard. Participants had the option to return the survey by mail or a URL was provided if they preferred to complete it online. Dentists were randomized to receive one of three incentives: $2 bill, customized pen/stylus, or no incentive. Results on this randomized experiment have been published previously [7], and full descriptive results from the survey, including the survey instrument itself, have been previously published [8].

Procedure provision was asked via the following survey question: “Given the differences between public and private insurance, we are interested in the types of services offered to DWP patients compared to privately insured patients. Please select the types of services you typically provide(d) to patients with DWP and with private insurance.” Types of services included the following: operative/restorative, endodontic (any), scaling and root planing, routine extractions, crown/bridge, removable partial dentures, and complete dentures.

Inclusion criteria for the study sample included general dentists who reported that they were either currently accepting new DWP patients or were not accepting new DWP patients but had treated more than 10 DWP patients in their practice in the previous six months. Subsequently in this article, we use the term “public insurance” to refer to the DWP.

Descriptive statistics were calculated for all service types and for respondents’ sociodemographic and practice characteristics. Sociodemographic and practice characteristics are provided for context to describe the study sample. Bivariate analyses using McNemar’s tests were performed to examine differences in dentist provision of services between privately and publicly insured patients for each service type. Statistical significance was set at p < 0.01.

Service provision patterns are presented for the full analytic sample. We also examined whether patterns varied by urbanicity. We hypothesized that low supply of dental specialists in rural areas may limit general dentists’ ability to refer patients out, particularly for patients with Medicaid. Urbanicity was measured using rural-urban commuting area (RUCA) codes, and classified practice locations as urban or rural based on the University of Washington Rural Health Research Center Categorization C [9]. It was determined by the University of Iowa Human Subjects Office that this project did not meet the regulatory definition of human subject research under a waiver approved by the Secretary, US Department of Health and Human Services, for Sect. 1115 projects conducted by the Centers for Medicare and Medicaid Services (CMS). The Dental Wellness Plan has federal approval via Sect. 1115 demonstration waiver. Informed consent was obtained via survey cover letter; completion and return of the survey indicated informed consent. All research activities were performed in accordance with ethical principles for research involving human subjects.

## Results

A total of 547 (43%) dentists responded to the survey, including 500 general dentists and 47 specialists. After excluding respondents who did not meet inclusion criteria (e.g., were not actively seeing any patients with Medicaid), the final analytical sample was 264 general private practice dentists. Table 1 provides a description of the sociodemographic and practice-related characteristics of the final sample. A majority of respondents were male, in solo practice, and worked 32 h/week or more. In terms of busyness, almost half were providing care to all requesting it, but did not feel overworked. Over half of responding dentists practiced in a rural area.

**Table 1:** Sociodemographic and practice-related characteristics of responding private practice general dentists in Iowa (N = 264)

Characteristics	n^†^(%)*
Age	
< 35 years	49 (19%)
35–44 years	59 (22%)
45–54 years	51 (19%)
55–64 years	55 (21%)
> 65 years	49 (19%)
Sex	
Male	174 (66%)
Female	90 (34%)
Practice arrangement	
Solo practice owner	138 (53%)
Partner	57 (22%)
Associate in the practice	40 (15%)
Employee in corporate owned practice	9 (3%)
Other arrangement	19 (7%)
Practice busyness	
Too busy to treat all requesting appointments	46 (18%)
Provided care to all requesting it, but felt overworked	75 (29%)
Provided care to all requesting it, but did not feel overworked	126 (48%)
Not busy enough, would like to have more patients	14 (5%)
Hours worked per week	
< 32 h	29 (11%)
>=32 h	235 (89%)
Urbanicity	
Urban	121 (46%)
Rural	143 (54%)

*Percentages may not sum to 100 due to rounding

^†^Variable ‘n’ may not sum to total due to missing observations

**Fig. 1:**
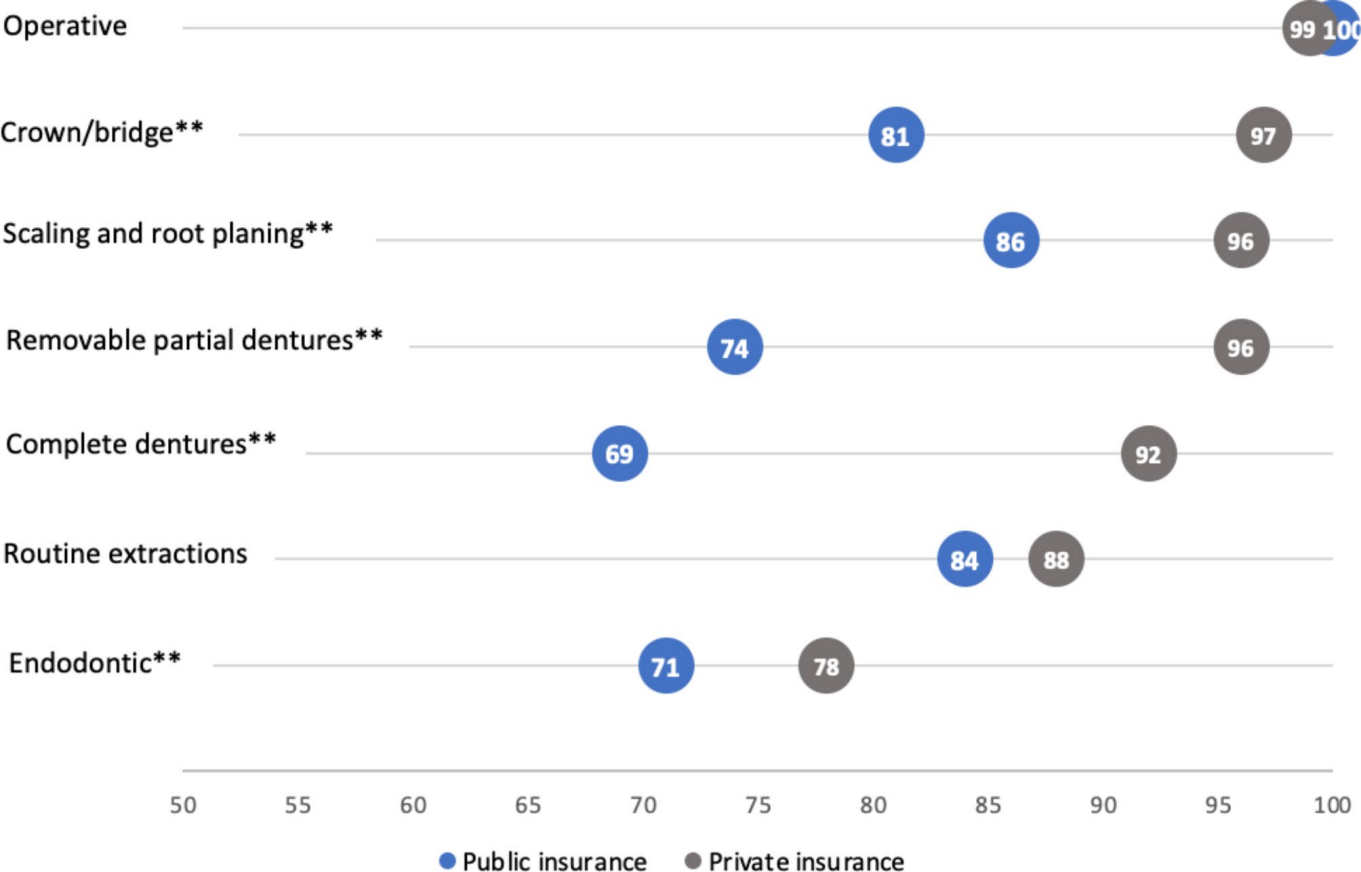
Percent of Iowa private practice general dentists providing types of dental services by patients’ dental insurance type (N = 264) **Significant at p < 0.001

Figure 1 shows the distribution of dental service categories provided, and compares response by insurance type. Dentists were significantly more likely to provide five of the seven types of services to patients with private insurance compared to patients with public insurance. The difference was most pronounced for complete dentures (Private: 92% vs. Public: 69%; p < 0.001), removable partial dentures (Private: 96% vs. Public: 74%; p < 0.001), and crown/bridge services (Private: 97% vs. Public: 81%; p < 0.001). Of all service categories, endodontic services were the least frequently provided for both insurance types (Private: 78% vs. Public: 71%; p < 0.001). Dentists’ reported provision of operative/restorative services and routine extractions were not significantly different by insurance type (p = 0.250 and p = 0.013, respectively).

**Fig. 2:**
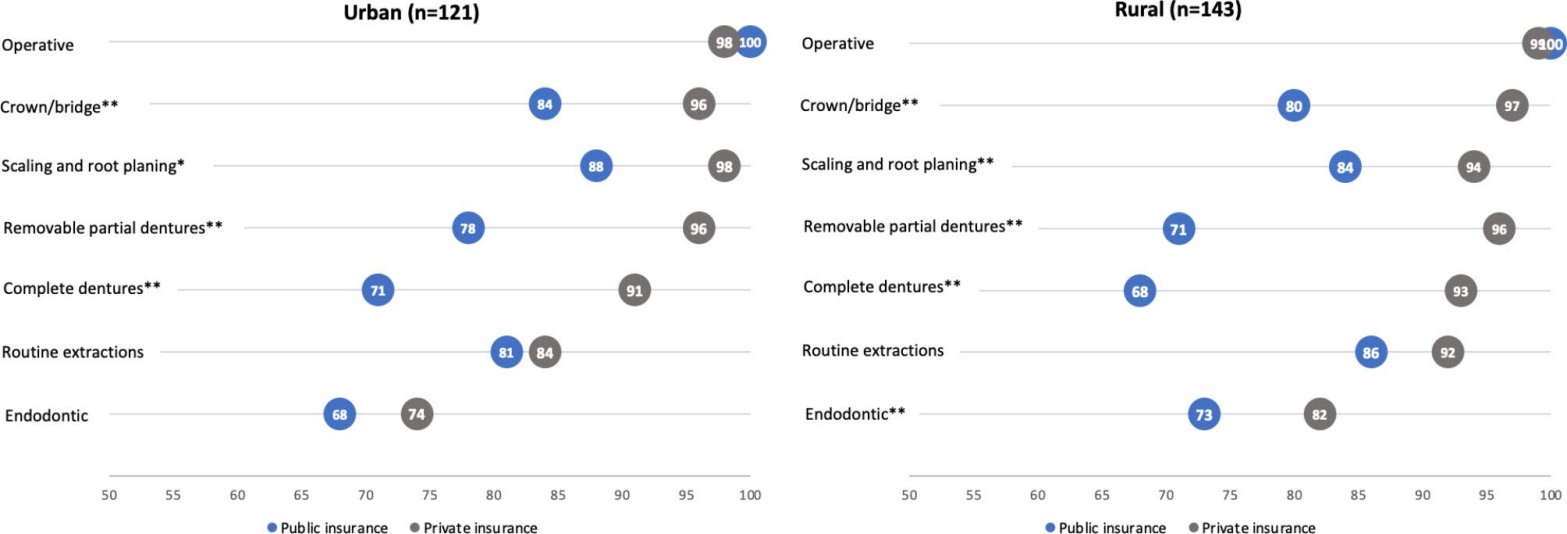
Percent of Iowa private practice general dentists providing types of dental services by patients’ dental insurance type and practice location *Significant at p < 0.01 **Significant at p < 0.001

Figure 2 shows provision of services by practice rurality. For both rural and urban providers, the greatest differences in service provision between publicly and privately insured patients was for complete and removable partial dentures, followed by crown/bridge and scaling and root planing. Among rural providers, there was a significant difference in the provision of endodontic services to privately versus publicly insured patients, whereas there was not a significant difference among urban providers for this category of services.

## Discussion

This study found significant differences in the types of services general dentists report providing to patients with private or public dental insurance. The three service types with the largest differences between groups were all prosthodontic services (e.g., complete dentures, removable partial dentures, and crown/bridge services). In previous reporting on this survey, respondent comments suggested that reimbursement for these types of services does not cover the cost of the lab fee, which may be driving lower provision of fixed and removable prosthodontic services to Medicaid-enrolled adults [8]. Additionally, among all survey respondents, 93% perceived reimbursement as a major problem in the program.

Low reimbursement is a well-known barrier to dentist Medicaid participation [10, 11]. One study found that, among 16 states that provided extensive Medicaid adult dental benefits, the average Medicaid reimbursement for adult dental services was approximately 37% relative to fees charged by dentists, and 46% relative to private dental insurance reimbursement [12]. However, there is significant variation within Medicaid program fee schedules with respect to how reimbursement for certain procedures compares to dentist fees, or to reimbursement from private insurers. For example, some states may purposefully reimburse preventive and routine restorative services at a higher rate relative to prosthodontic services, to encourage receipt of the most basic dental services. To the authors’ knowledge, no studies have examined within-state variation in relative reimbursement and potential impact on treatment patterns. The relative importance that a state assigns to different types of dental care via reimbursement rates may influence the services dentists provide to patients with Medicaid. However, it is increasingly difficult to study this phenomenon given the increasing role of dental managed care organizations in state Medicaid dental programs as fee schedules are less often publicly available, and may or may not reflect the state’s priorities for receipt of certain types of care.

The analytic sample in this study (n = 264) was composed of dentists who were currently accepting patients with Medicaid or had treated at least 10 patients with Medicaid in the last 12 months, which was approximately half (48%) of the 547 survey respondents. This is not far from an estimate from the American Dental Association Health Policy Institute, which found that a total of 61% of professionally active dentists in Iowa (including all dentists, not just those in private practice) had treated at least 10 child patients with Medicaid in the last year [13].

Limitations in this study include the potential biases inherent in survey research, particularly social desirability bias as participants may have been inclined to report more equity in the types of services provided to privately and publicly insured populations if they suspected that it would be viewed more favorably by others. Response bias could also affect results if Medicaid-participating survey respondents differed systematically in their practice patterns from Medicaid-participating dentists who did not participate in the survey. We were not able to assess this as we did not have participation information on non-respondents. Additionally, this study was conducted in one state and results may not be generalizable to other states with varying degrees of Medicaid participation and different Medicaid dental plan structure and reimbursement.

This study contributes to the literature regarding the nuance in dentist participation in Medicaid. While participation is often described as a binary outcome (e.g., a provider participates or does not), previous studies have found that many participating dentists place limits on participation, such as a set number of patients per month or only accepting referrals from other providers [5]. This study identified further nuance in the types of services provided to this population relative to privately insured patients. This differential could impact oral health disparities if patients with Medicaid do not have the same access to rehabilitative services, such as complete or partial dentures to replace missing teeth, as privately insured patients do since these services affect functional status and satisfaction with appearance. Future research should examine the potential roles of dentist and practice characteristics on service provision, service provision to publicly insured populations in other states, and the impact of state Medicaid program fee schedules on service provision for this population.

## Data Availability

The datasets generated and/or analyzed during the current study are not publicly available because they are part of a CMS Sect. 1115 waiver evaluation but full descriptive survey results are available from the corresponding author on request.
